# Vital pulp therapy using Biodentine XP and adhesive restorations: a three-year case series

**DOI:** 10.3389/fdmed.2026.1837786

**Published:** 2026-07-29

**Authors:** Vincenzo Tosco, Riccardo Monterubbianesi, Francesco Puleio, Giulia Orilisi, Flavia Vitiello, Angelo Putignano, Giovanna Orsini

**Affiliations:** 1 Department of Life Sciences, Health and Health Professions, Link Campus University, Rome, Italy; 2 Department of Clinical Sciences and Stomatology, Università Politecnica Delle Marche, Ancona, Italy; 3 Department of Biomedical and Dental Sciences and Morphofunctional Imaging, Messina University, Messina, Italy

**Keywords:** vital pulp therapy, direct pulp capping, dental pulp exposure, calcium silicate–based cements, bioactive dental materials, adhesive restoration, clinical case series, pulp preservation

## Abstract

Vital pulp therapy (VPT) has become a predictable biologically driven alternative to root canal treatment for the management of deep carious lesions and limited pulp exposures, particularly with the advent of hydraulic calcium silicate cements (hCSCs). The novelty of this case series lies in the use of Biodentine XP as a bioactive dentin substitute combined with immediate or staged adhesive restorative protocols in posterior permanent teeth, with a 3-year clinical and radiographic follow-up. Three patients presenting with deep carious lesions and signs consistent with reversible pulpitis were treated under rubber dam isolation, involving a total of four posterior teeth. Direct pulp capping was performed in two patients, respectively in a mandibular premolar (Case 1) and a mandibular first molar (Case 2) after pulp exposure during caries removal, whereas indirect pulp capping was performed in two mandibular molars of the same patient (Case 3) affected by deep carious lesions. Diagnosis was established through clinical examination, pulp sensibility testing, percussion, and periapical radiographic assessment, which confirmed deep dentinal involvement without apical disease. After complete caries removal and hemostasis assessment when required, Biodentine XP was applied using a bio-bulk fill approach. In the direct pulp capping cases, treatment was completed in a single visit with immediate adhesive composite restoration using a 10-MDP–based universal adhesive. In the indirect pulp capping case, Biodentine XP was initially used as a temporary restoration and subsequently maintained as a definitive dentin substitute during final direct or indirect adhesive rehabilitation. At 6 months, 1 year, and 3 years, all treated teeth remained asymptomatic and responsive to pulp sensibility testing, while periapical radiographs showed stable conditions without signs of pathology or treatment failure. Restorations also demonstrated satisfactory marginal integrity and structural stability over time. These cases suggest that a bioactive–adhesive approach based on rapid-setting hCSCs and immediate or staged coronal sealing may represent a clinically reliable and minimally invasive strategy for preserving pulp vitality in selected posterior teeth. Given the descriptive nature of this three-case series, these findings should be interpreted with caution and require confirmation through larger prospective studies.

## 1 Introduction

Vital pulp therapy (VPT) is increasingly used as a minimally invasive approach for managing deep carious lesions and limited pulp exposures in permanent teeth. Its objective is to preserve the vitality and defensive function of the dentin–pulp complex whenever the clinical condition remains compatible with repair. The long-term outcome of VPT depends on appropriate case selection, control of pulpal bleeding, and durable coronal sealing, since bacterial leakage may compromise pulp survival even when the initial biological response is favorable (1–8). Calcium hydroxide represented the traditional reference material for many years, but its limitations in terms of solubility, sealing ability, and long-term stability reduced its predictability (9). The introduction of mineral trioxide aggregate (MTA), one of the earliest and most widely used hydraulic calcium silicate–based cements (hCSCs), improved biological performance and stimulated renewed interest in conservative pulp management, while more recent hCSC formulations have further strengthened this field by combining bioactivity with more favorable operative characteristics (10, 11). These materials create an alkaline environment, release calcium ions, and support interfacial mineralization, thereby contributing to pulp protection and reparative hard tissue deposition (12). Among these materials, Biodentine has been widely investigated as a dentin substitute because of its biocompatibility, bioactivity, and relatively short setting time (13). Biodentine XP was introduced as a formulation that maintains the same biological rationale and established profile as conventional Biodentine, while providing more standardized consistency and simplified handling through mechanical activation, automatic mixing, and capsule-based delivery (14, 15). In the present report, Biodentine XP was selected for these workflow-related characteristics rather than for any presumed superiority over previous formulations. When a bio-bulk fill approach is adopted, the hCSC is used not only for pulp protection, but also as a dentin substitute intentionally maintained directly beneath the definitive restoration. As a result, the material becomes part of the adhesive-restorative interface rather than simply a pulp-capping layer. Under these conditions, the adhesive strategy becomes clinically relevant because the hCSC may still exhibit limited surface hardness during its early setting phase and may therefore be more susceptible to disruption by acids, solvents, or manipulation. For this reason, immediate restoration over hCSCs may require either a short waiting period, a protective intermediate layer, or adhesive systems able to interact favorably with calcium silicate substrates, in order to preserve interface continuity and ensure durable coronal sealing (16). Universal adhesives containing functional monomers such as 10-MDP may enhance the interaction between resin-based restorative materials, dentin, and calcium silicate surfaces, supporting this integrated restorative approach (17, 18). The present case series reports three patients and four treated posterior teeth with deep carious lesions, clinical findings consistent with reversible pulpitis, and no radiographic evidence of apical disease. The cases were selected on the basis of symptom assessment, pulp sensibility testing, percussion findings and periapical radiographic examination. Two teeth were treated with direct pulp capping after pulp exposure during caries removal, whereas one indirect pulp capping case involved two posterior teeth restored according to different treatment timings. The added clinical value of this report lies in the documented use of Biodentine XP within different adhesive restorative workflows, including immediate and staged protocols as well as direct and indirect rehabilitation, all followed for 3 years. The primary aim of this case series was therefore to describe the clinical integration of Biodentine XP with contemporary adhesive restorative protocols in VPT and to report the associated 3-year clinical and radiographic outcomes.

## 2 Case reports

This article reports retrospective descriptive clinical cases derived from routine, non-experimental care, without study-specific interventions beyond standard practice. The manuscript was prepared in accordance with the ethical principles of the Declaration of Helsinki (2024 version) and in line with the CARE guidelines (Supplementary Table S1). The main demographic, diagnostic, therapeutic, and follow-up data of the included cases are summarized in Table 1. Written informed consent for treatment was obtained from all patients before the procedures. In addition, all patients provided specific written consent for the anonymized publication of clinical photographs and radiographs.

**Table 1:** General clinical data of 3 cases. Demographic data, clinical characteristics, treatment protocols, and quantitative outcomes of the included VPT cases. Outcomes include electric pulp test (EPT) values recorded at baseline and at 3-year follow-up, periapical index (PAI) scores derived from periapical radiographic evaluation (Rx).

Case No.	Gender	Age (years)	Diagnosis	Clinical Condition	Symptoms	Treatment	Outcomes	Follow-up
1	Female	32	Deep carious lesion	Mandibular second premolar–mechanical pulp exposure after caries removal	No spontaneous pain	Direct pulp capping with Biodentine XP and composite restoration after the setting of the material (single session)	EPT: 25 *μ*A → 31 μA; PAI (Rx) = 0	6 months 1 year 3 years
2	Female	17	Reversible pulpitis	Mandibular first molar – mechanical pulp exposure during caries removal	Prolonged sensitivity to cold, stimulus-dependent pain, no spontaneous or lingering pain	Direct pulp capping with Biodentine™ XP and immediate composite restoration (single session)	EPT: 27 μA → 33 μA; PAI (Rx) = 0	6 months 1 year 3 years
3	Male	40	Reversible pulpitis	Indirect pulp capping with Biodentine XP – second molar: staged indirect overlay restoration; third molar: delayed direct composite restoration	Prolonged sensitivity to cold, no spontaneous or lingering pain	Indirect pulp capping with Biodentine™ XP – second molar: immediate restoration; third molar: two-step approach with delayed restoration	EPT: 29 μA → 30 μA; PAI (Rx) = 0	6 months 1 year 3 years

This case series reports three patients treated with VPT in posterior permanent teeth and followed for 3 years. From January 2023 to April 2026, three patients in good systemic health, aged between 17 and 40 years, were treated using VPT. All cases showed clinical and radiographic findings consistent with reversible pulpitis. Symptom presentation differed slightly among patients, ranging from stimulus-dependent cold sensitivity to the absence of spontaneous pain. In all cases, deep carious lesions were detected clinically and radiographically, pulp sensibility tests (cold and electric) elicited positive responses, and periapical radiographs showed no evidence of apical pathology, internal resorption, or calcific metamorphosis. Before treatment, all patients were informed about the rationale for a biologically oriented, vitality-preserving approach as an alternative to conventional root canal treatment whenever clinical conditions were favorable. The potential benefits of maintaining pulp vitality, as well as the possible risk of treatment failure and the need for subsequent endodontic intervention, were clearly discussed.

In direct pulp capping cases, bleeding control was assessed clinically after gentle compression with a sterile saline-moistened pellet; continuation of the procedure required complete hemostasis within 5 min and absence of uncontrolled pulpal bleeding. At follow-up, restorative performance was assessed according to modified United States Public Health Service (USPHS)-based criteria, including marginal adaptation, marginal discoloration, secondary caries, and retention/fracture events (19). Scores were classified as A (clinically acceptable), B (clinically unacceptable but not requiring replacement), or C (clinically unacceptable and requiring replacement). Detailed restoration survival outcomes for each treated tooth are reported in Supplementary Table S2. Radiographic evidence of hard tissue deposition was assessed descriptively on serial periapical radiographs, in line with the radiographic follow-up approach previously adopted in our earlier clinical case series (19); however, no software-based quantitative assessment or standardized grading system was applied in the present report.

### 2.1 Case 1

A 32-year-old female patient presented requesting treatment for a “hole in her tooth.” She did not report pain. Pulp sensibility testing was positive, while percussion testing was negative. After discussion of the available therapeutic options and the expected prognosis associated with VPT, written informed consent was obtained.

**Figure 1:**
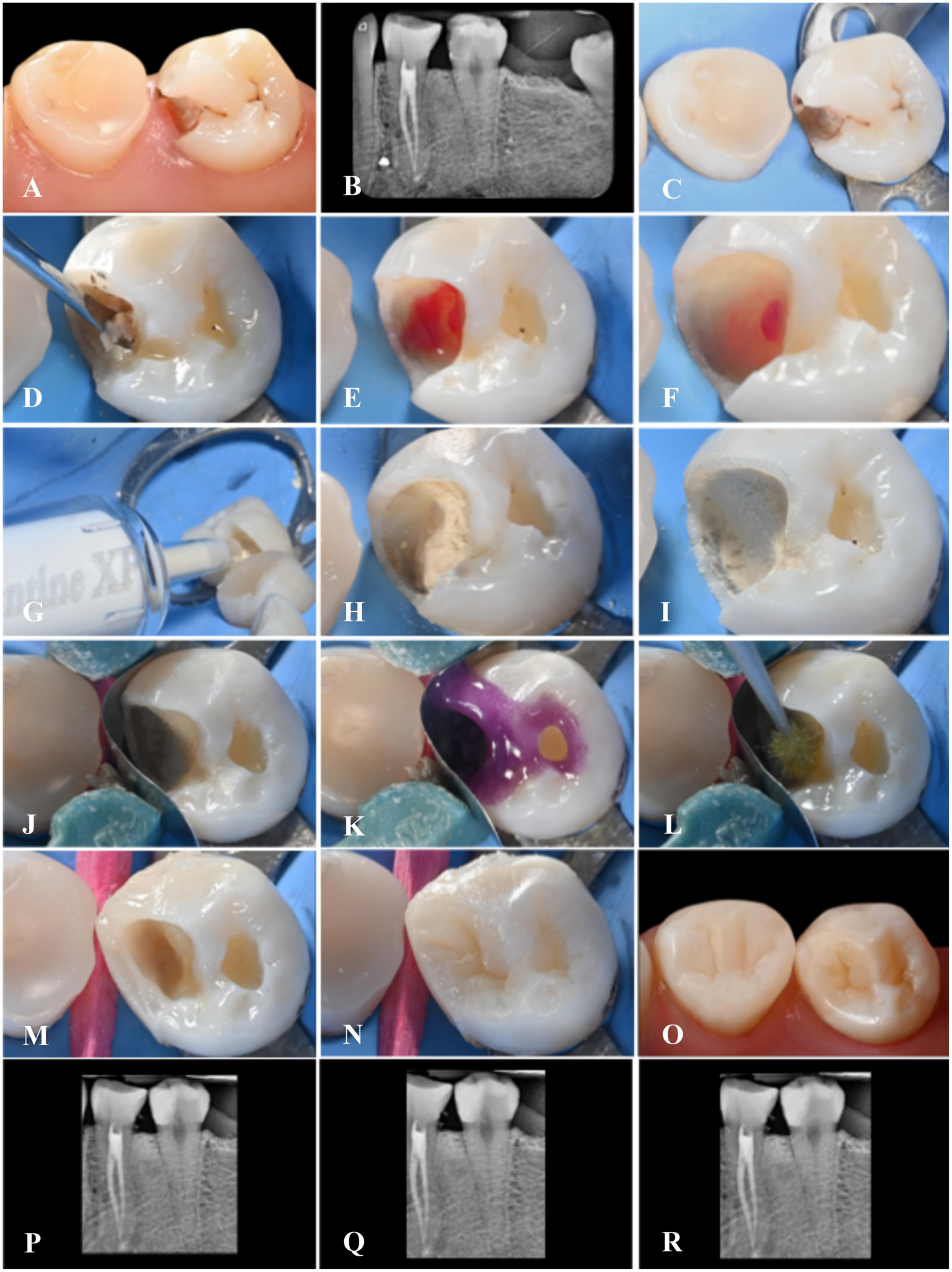
Step-by-step workflow of Case 1 (direct pulp capping). **(A,B)** Preoperative clinical and radiographic views. **(C–F)** Cavity access and caries removal, pulp exposure, and hemostasis. **(G–I)** Placement of Biodentine XP. **(J–O)** Adhesive procedures and definitive composite restoration. **(P–R)** Periapical radiographs at 6 months **(P)**, 1 year **(Q)**, and 3 years **(R)** follow-up.

Clinical examination revealed a deep carious lesion on the mandibular left second premolar, without visible pulp exposure (Figure 1A). The preoperative radiograph confirmed the depth of the lesion and its proximity to the pulp chamber, while showing an intact lamina dura and no signs of periapical involvement (Figure 1B). Pulp sensibility testing elicited a normal, non-lingering response, supporting a diagnosis of reversible pulpitis associated with a deep carious lesion.

After rubber dam placement, the cavity was accessed using both high- and low-speed burs to remove the bulk of the carious tissue (Figure 1C). As the excavation approached the pulp, manual instruments, including a vanadium excavator, were used to improve tactile control and selectively remove infected dentin (Figure 1D). During the final stage of excavation, a clinically small pulp horn exposure was observed in the deepest portion of the cavity; no calibrated measurement of the exposure size was performed (Figure 1E). Hemostasis was achieved by gentle compression with a sterile pellet moistened with physiological saline until complete bleeding control was obtained, indicating a favorable pulpal response and allowing continuation of the procedure (Figure 1F). In the presence of controlled bleeding, a direct pulp capping procedure was performed. Biodentine XP was applied directly over the exposed pulp and used to fill the deepest portion of the cavity as a bioactive dentin substitute beneath the definitive restoration, according to a bio-bulk fill approach (Figure 1G). The material was allowed to set for approximately 15 min before adhesive procedures were initiated, ensuring adequate surface hardness (Figure 1H). After initial setting, the Biodentine XP surface was refined with burs and hand instruments to obtain a uniform and well-adapted base (Figure 1I). A sectional matrix was then placed to convert the Class II cavity into a Class I configuration (Figure 1J), and selective enamel etching was performed with 37% phosphoric acid for 20 s (Figure 1K). A universal adhesive (Scotchbond Universal Plus, Solventum) was then applied, with no displacement of the Biodentine XP surface, confirming adequate material hardening (Figure 1L). The mesial wall was rebuilt to establish proper proximal contact (Figure 1M), and the cavity was restored with a single increment of bulk-fill composite resin (One Bulk Fill Restorative, Solventum). Occlusal anatomy was shaped according to the Essential Lines technique (Figure 1N) (20). A glycerin gel barrier was applied, followed by final light curing. After rubber dam removal, occlusion was checked and finishing and polishing procedures were completed immediately in the same visit (Figure 1O) (21).

At the immediate postoperative evaluation, the restoration showed appropriate anatomical contour and occlusal integration, with no postoperative discomfort and normal responses to pulp sensibility testing. At the 6-month follow-up, the tooth remained asymptomatic, vitality was preserved, and the radiograph showed an intact lamina dura, no periapical changes, and stable adaptation of the calcium silicate–based capping material (Figure 1P). After 1 year, the patient remained symptom-free, pulp sensibility testing was still positive, and radiographic examination confirmed periapical health together with early evidence of hard tissue deposition beneath the capping layer (Figure 1Q). At the 3-year assessment, pulp vitality was maintained, with normal clinical responses and no signs of internal or external resorption. Radiographically, periodontal support remained stable and a well-defined dentinal bridge was visible, supporting the long-term clinical and radiographic success of the procedure (Figure 1R).

### 2.2 Case 2

**Figure 2:**
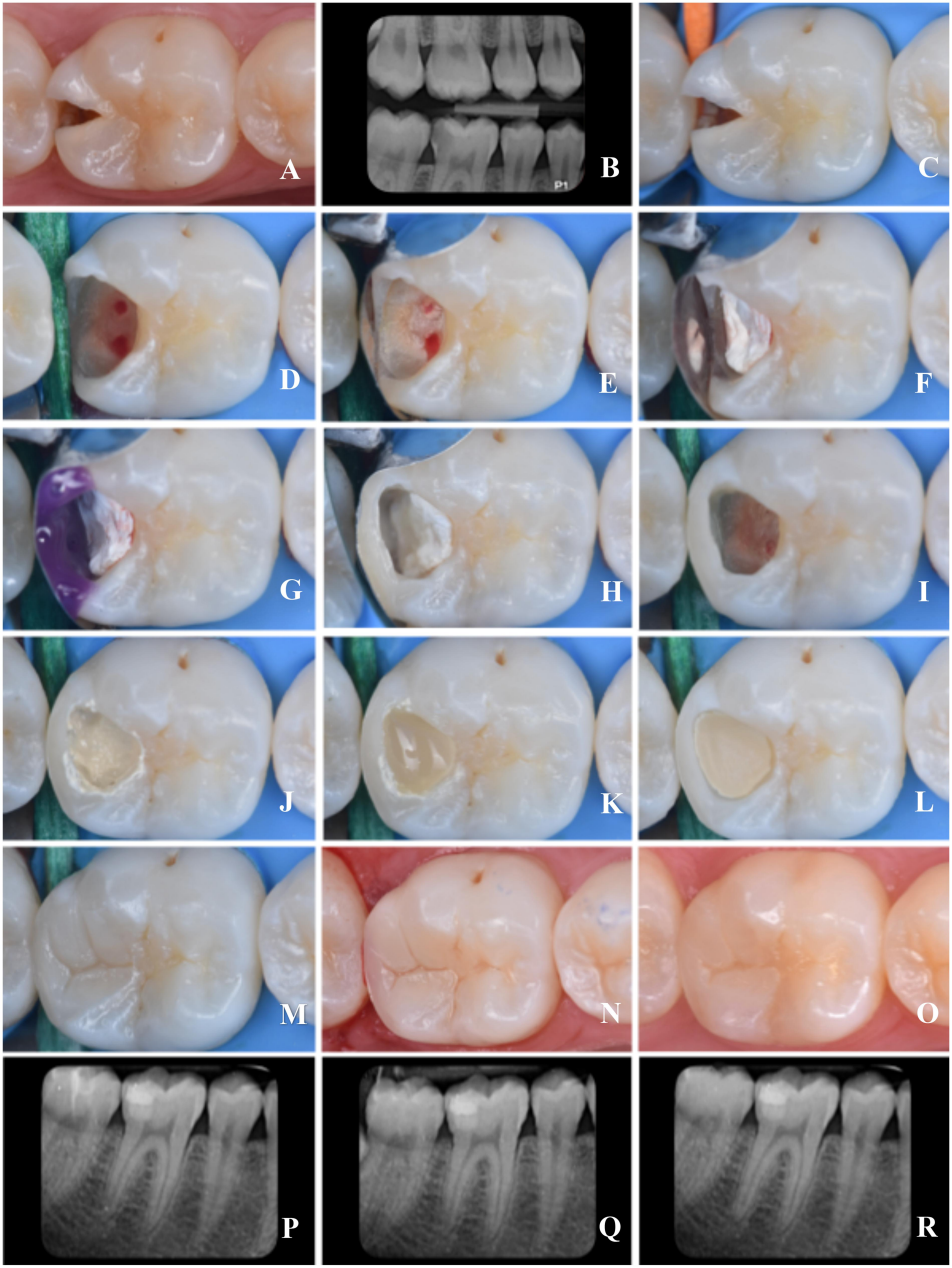
Step-by-step workflow of Case 2 (direct pulp capping). **(A,B)** Preoperative clinical and radiographic views. **(C–F)** Cavity access and caries removal, pulp exposure, and hemostasis. **(G–J)** Reconstruction of the distal wall and placement of Biodentine XP. **(K–O)** Immediate adhesive procedures and definitive Class I composite restoration, carried out without waiting for the complete setting of Biodentine XP. **(P–R)** Periapical radiographs at 6 months **(P)**, 1 year **(Q)**, and 3 years **(R)** follow-up.

A 17-year-old female patient presented with stimulus-dependent cold sensitivity localized to the mandibular first molar, which resolved immediately after removal of the stimulus, with no spontaneous or lingering pain. Clinical examination revealed a deep distal carious lesion (Figure 2A), while radiographic assessment confirmed a D3 lesion according to the American Dental Association radiographic caries classification (Figure 2B). The tooth responded positively to cold and electric pulp sensibility tests and negatively to percussion, and no radiographic signs of periapical pathology were observed. These findings supported a diagnosis of reversible pulpitis associated with deep dentinal caries. After discussion of the treatment options and the rationale for vital pulp therapy, written informed consent was obtained. Following rubber dam isolation, a wooden wedge was placed interproximally to stabilize the rubber dam and slightly separate the teeth, thereby improving access to the distal margin (Figure 2C). Cavity preparation was initiated with high-speed burs to remove the superficial infected tissue, followed by low-speed excavation as the preparation approached the deeper carious layers. Although a hard-dentin endpoint was reached, the remaining dentin still appeared clinically compromised, and during the final stages of excavation, two pulp horns became mechanically exposed, resulting in a Class II cavity configuration (Figure 2D).

To allow controlled management of the pulp exposure and facilitate the restorative procedure, an interproximal matrix and separation ring were placed to rebuild the proximal wall before direct pulp capping. This converted the cavity into a Class I configuration, providing a more enclosed and stable environment for placement of the hCSC (Figure 2E). Before the adhesive steps required for proximal wall reconstruction, the exposed pulp horns were covered with a sterile pellet to allow spontaneous hemostasis, while sterile PTFE tape was used to isolate and protect the pulp tissue from phosphoric acid and adhesive contamination (Figure 2F). Selective enamel etching was then performed on the distal margin with 37% phosphoric acid for 20 s (Figure 2G), after which the distal wall was rebuilt with composite resin to re-establish the correct contour and contact point (Figure 2H). Once the matrix and ring were removed, a Class I cavity configuration was obtained, the PTFE tape was removed, and complete hemostasis was confirmed (Figure 2I).

A direct pulp capping procedure was then performed using Biodentine XP applied with a bio-bulk fill technique (Figure 2J). The material was gently adapted with a brush to seal the exposed pulp horns and cover the deepest portion of the cavity. Immediately after placement of the hydraulic calcium silicate cement, and without waiting for complete setting, a self-adhesive flowable composite (Vertise Flow, Kerr Corporation, Orange, CA, USA) was applied as a protective surface layer and light-cured (Figure 2K). In this specific case, the self-adhesive flowable was not intended to replace a 10-MDP–based universal adhesive in terms of long-term adhesive integration, but was used as an intermediate protective layer to allow completion of the restoration in a single appointment, without waiting for complete setting of Biodentine XP. This approach was selected to obtain rapid coronal sealing and, at the same time, to protect the freshly placed bioactive material during its early setting phase, reducing the risk of disturbing its interaction with the dentin substrate during the subsequent adhesive steps. However, the possibility of lower long-term interfacial stability compared with direct adhesive integration using a 10-MDP universal adhesive was recognized. The cavity margins were then refined with a finishing bur, beveled, and prepared for the adhesive protocol (Figure 2L). A universal adhesive was actively scrubbed onto the substrate, gently air-thinned, and light-cured. The final restoration was completed with a body-shade composite resin, sculpted according to the Essential Lines technique to reproduce the occlusal anatomy (Figure 2M). After rubber dam removal, occlusion was adjusted and the restoration was finished and polished to achieve a smooth and anatomically refined surface (Figure 2N).

At the 6-month follow-up, the tooth remained completely asymptomatic, with normal responses to pulp sensibility testing and negative percussion. Clinically, the composite restoration was intact, with no signs of marginal discoloration, wear, or infiltration (Figure 2O). Radiographic evaluation showed stable periapical conditions, an intact periodontal ligament space, and no evidence of secondary caries or coronal leakage. The radiograph also clearly distinguished the hydraulic calcium silicate cement from the overlying composite, confirming satisfactory adaptation and marginal integrity. At the 1-year recall, the tooth continued to respond positively to vitality testing, and radiographic examination revealed early signs of hard tissue deposition beneath the capping material, consistent with initial dentinal bridge formation. At the 3-year follow-up, pulp vitality was still maintained, symptoms were absent, and radiographic examination showed a well-defined dentinal bridge together with a stable composite restoration.

### 2.3 Case 3

**Figure 3:**
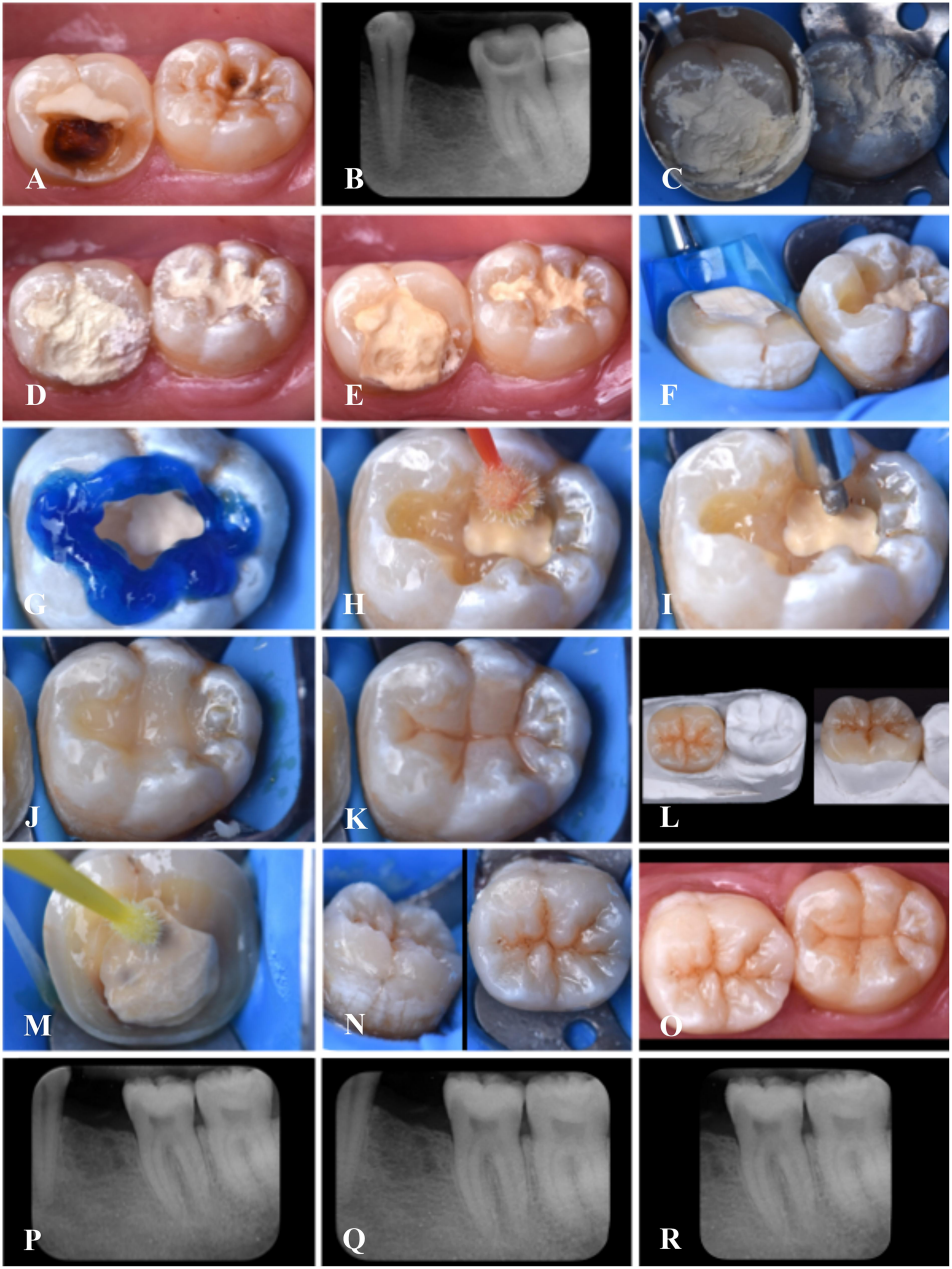
Step-by-step workflow of Case 3 (indirect pulp capping). **(A,B)** Preoperative clinical and radiographic views. **(C–E)** Cavity access and caries removal, Biodentine XP application as temporary filling in bio-bulk fill approach. **(F–K)** Preparation of indirect restoration on second molar and direct restoration performed on third molar. **(L–O)** Cementation of indirect restoration. **(P–R)** Periapical radiographs at 6 months **(P)**, 1 year **(Q)**, and 3 years **(R)** follow-up.

A 40-year-old male patient presented on an emergency basis reporting a fractured restoration and cold-induced discomfort in the left mandibular posterior region. Clinical examination revealed an old defective restoration on the mandibular second molar, associated with a fractured lingual wall related to a deep carious lesion. The adjacent third molar also exhibited an occlusal carious lesion (Figure 3A). The patient reported stimulus-dependent sensitivity without spontaneous or lingering pain. Both teeth responded positively to pulp sensibility testing and negatively to percussion, indicating preserved pulp vitality and absence of periapical symptoms.

Preoperative radiographs confirmed deep caries beneath the failed restoration on the second molar and showed an intact periodontal ligament space, with no signs of periapical pathology or root resorption (Figure 3B). The third molar also showed deep carious involvement without radiographic evidence of apical disease. Based on the patient's symptoms and the clinical–radiographic findings, a diagnosis of reversible pulpitis associated with deep caries was established for both teeth. After discussion of the treatment plan, restorative options, and expected prognosis, written informed consent was obtained.

Following rubber dam isolation, caries excavation and cavity cleaning were carried out on both teeth. Biodentine XP was selected as the hCSC to be used as a pulp-protective dentin substitute. The material was placed in both cavities using a bio-bulk fill technique and allowed to set for approximately 12 min, providing immediate sealing and allowing biological monitoring before definitive rehabilitation (Figure 3C). After removal of the automatrix and rubber dam, the integrity of the temporary seal was confirmed clinically (Figure 3D).

At the 1-month recall, slight occlusal wear of the temporary Biodentine XP restorations was observed, while pulp vitality remained preserved in both teeth, confirming the suitability of the material as a dentin substitute during the interim phase, although not as a definitive occlusal restorative material (Figure 3E). At this stage, the choice between immediate and delayed definitive rehabilitation was based on the extent of residual tooth structure loss and the expected restorative complexity. The third molar, which allowed a direct adhesive restoration after interim pulpal monitoring, was restored directly. In contrast, the second molar presented greater structural compromise and required cuspal coverage with an indirect overlay; therefore, a staged restorative approach was selected.

After renewed rubber dam isolation, the LM Gengiva instrument (LM-Dental, Parainen, Finland) was used to retract the dam apically at the lingual margin, improving access to the preparation area (Figure 3F). The second molar was prepared according to smoothed structural design principles, including rounded internal angles, continuous surface transitions, and cusp reduction directed toward the axial wall to improve stress distribution and reduce fracture risk. Throughout the preparation phase, the Biodentine XP base remained stable and mechanically intact, showing adequate hardness to serve as a reliable dentin substitute and support the indirect restorative workflow.

For the third molar, the Class I cavity was refined while preserving the underlying Biodentine XP up to approximately 1.5 mm from the occlusal surface. Selective enamel etching of the margins was performed with 37% orthophosphoric acid for 20 s (Figure 3G). A 10-MDP–based universal adhesive (Scotchbond Universal Plus, Solventum, St. Paul, MN, USA) was then applied with active scrubbing, followed by air-drying for 20 s and light curing (Figure 3H). After verifying adequate space for the final enamel layer with the LM Misura Posterior instrument (LM-Dental, Parainen, Finland) (Figure 3I), a single mass of body-shade composite resin was placed to complete the direct restoration (Figure 3J). The final anatomy was developed according to the Essential Lines technique, and the occlusal grooves were characterized with brown stains (Figure 3K).

At the subsequent appointment, once the indirect overlay for the second molar was available for cementation (Figure 3L), the temporary restoration was removed and the tooth surface was cleaned by sandblasting. The underlying Biodentine XP remained intact and well adapted beneath the preparation. The adhesive procedures were then performed following the same strategy used for the third molar (Figure 3M). The overlay was luted using RelyX Universal (Solventum, St. Paul, MN, USA) adhesive resin cement according to the step luting technique (22). Seating and excess removal were assisted by the LM Condensa and LM Eccesso instruments (LM-Dental, Parainen, Finland), respectively, to improve adaptation and cement cleanup (Figure 3N). After rubber dam removal, all margins were finished and polished, and occlusion was checked and adjusted as required (Figure 3O).

At the 6-month follow-up, the patient was completely asymptomatic, with normal responses to pulp sensibility testing and negative percussion findings on both molars. Radiographic assessment demonstrated stable periodontal and periapical structures, as well as well-adapted restorations without evidence of coronal leakage, marginal breakdown, or secondary caries (Figure 3P). Long-term evaluations performed at 1 year (Figure 3Q) and 3 years (Figure 3R) confirmed maintenance of pulp vitality, absence of symptoms, and radiographic stability in both teeth.

Across all treated teeth, restorative performance remained clinically acceptable at the 3-year follow-up. No secondary caries, loss of retention, or fracture events were observed, and marginal adaptation and discoloration remained within clinically acceptable limits. Detailed restoration survival outcomes for each treated tooth are reported in Supplementary Table S3.

## 3 Discussion

VPT is now widely regarded as a biologically oriented strategy for the management of deep carious lesions and limited pulp exposures in permanent teeth. In the present case series, treatment planning was guided primarily by pulpal diagnosis, intraoperative bleeding control, and the feasibility of achieving a stable long-term coronal seal, rather than by patient age alone (23). The present series also supports the concept that case selection remains the main determinant of VPT success, whereas the properties of the pulp-capping material and restorative interface should be regarded as supporting factors rather than the sole explanation for outcome. This rationale supported the management of Cases 1 and 2 with direct pulp capping followed by immediate adhesive restoration, whereas Case 3 was treated with indirect pulp capping in a staged restorative workflow. From this perspective, the present series is of particular interest because all treated teeth remained asymptomatic and vital throughout the 3-year follow-up, while also illustrating how a next-generation hCSC may be integrated with contemporary adhesive restorative procedures in different clinical scenarios. In teeth with pulp exposure, including mechanical exposures occurring during caries removal, direct pulp capping may achieve favorable and predictable outcomes in permanent teeth when pulp vitality is confirmed and bleeding can be controlled (24). In the present series, this rationale guided the management of Cases 1 and 2, in which direct pulp capping was performed after confirmation of pulp vitality and successful hemostasis. In both cases, a single-visit restorative approach was selected in order to provide immediate sealing of the cavity, reduce the risk of bacterial contamination, and protect the exposed pulp through rapid definitive restoration (25–27). By contrast, deep carious lesions with minimal residual dentin thickness remain among the most challenging scenarios for pulp preservation. In such cases, indirect pulp capping may offer a conservative strategy to protect the pulp while preserving residual tooth structure. In Case 3, Biodentine XP was used not only as a pulp-protective material, but also as a bioactive dentin substitute during a staged restorative workflow. Under these conditions, the material may contribute to reducing dentin permeability, supporting mineral deposition at the dentin–pulp interface, and preserving structural support for subsequent adhesive rehabilitation Indeed, the most original aspect of the present series lies in the interaction between Biodentine XP, universal adhesive systems, and resin-based composite restorations. In general, the long-term success of VPT depends not only on the biological response of the pulp, but also on the quality of the restorative interface and the durability of the coronal seal. In this sense, the present cases suggest that Biodentine XP can be incorporated into a broader restorative concept in which pulp protection and definitive rehabilitation are part of the same treatment strategy. This interpretation is consistent with previous clinical observations, where favorable VPT outcomes were closely associated with the quality of the final restoration and not exclusively with the pulp-capping material itself (19, 28). A central point emerging from these cases is the adhesive integration between Biodentine XP and resin-based restorative materials. Within the limitations of this case series, however, it is not possible to determine the relative contribution of the Biodentine XP–adhesive interface versus the peripheral resin–enamel seal to the favorable clinical outcomes observed. The maintenance of a durable coronal seal, particularly at the enamel–restoration interface, should also be considered a substantial contributing factor in limiting bacterial leakage and supporting pulp preservation. Therefore, the interaction between Biodentine XP and adhesive restorative materials should be interpreted as a biologically and technically plausible supporting factor within an integrated restorative strategy, rather than as a proven primary determinant of success. Current evidence suggests that long-term interface stability is strongly influenced by the interaction between calcium-rich Biodentine surfaces and functional phosphate monomers such as 10-MDP (27, 29). The phosphate groups of 10-MDP may partially dissolve and recrystallize the cement surface, generating a nano-layered calcium–phosphate structure that reinforces the interface and supports long-term sealing. For this reason, universal adhesives containing 10-MDP were selected in the present cases to improve integration, mechanical continuity, and biological protection between Biodentine XP and the resin-based composite restoration. In selected clinical situations, *in vitro* evidence supports the use of self-adhesive flowable composites containing acidic functional monomers directly over freshly placed Biodentine XP as a time-efficient strategy to obtain an early coronal seal (30). Acting as an intermediate protective layer, this material may shield the hCSC from acids or solvents during subsequent adhesive procedures, thereby allowing continuation of treatment without directly disturbing the surface of the bioactive cement during its early setting phase (31). In Case 2, this approach was adopted for a specific clinical reason: to complete the restoration in a single appointment, achieve rapid coronal sealing, and protect freshly placed Biodentine XP while preserving workflow continuity. However, this strategy should not be interpreted as equivalent to direct 10-MDP–based adhesive integration in terms of long-term interfacial performance. Rather, it should be regarded as a pragmatic option for selected situations in which immediate sealing is prioritized, while acknowledging that further studies and longer clinical follow-up are needed to better assess its long-term feasibility. Conversely, glass ionomer cements are not advised as permanent covering materials because they lack chemical affinity with Biodentine and demonstrate inferior sealing performance (29). Overall, current evidence supports the combined use of hCSC and functional monomers such as 10-MDP as a predictable strategy for creating a stable, integrated interface capable of sustaining the long-term success of VPT (17, 32). Within this perspective, the main clinical value of Biodentine XP in the present series lies in its dual role: as a bioactive material capable of supporting pulp preservation and as a dentin substitute that can be incorporated into contemporary adhesive restorative protocols. Nevertheless, despite these encouraging findings, important knowledge gaps remain. As a small clinical case series, this report provides limited evidence and restricted generalizability. In addition, a degree of selection bias cannot be excluded, since the report includes only a limited number of successful clinical cases. Although the series includes three clinical cases with a minimum 3-year follow-up, the findings should be regarded as preliminary clinical evidence requiring confirmation through larger, multicenter prospective studies. Outcome assessment relied on clinical examination, pulp sensibility testing, patient-reported symptoms, and periapical radiographs, while advanced imaging and laboratory analyses were not performed. Consequently, dentin bridge formation and microleakage at the various interfaces were not directly assessed, and the radiographic findings should be interpreted as indicative rather than as definitive evidence of barrier formation in hard tissues. In addition, pulp sensibility tests have intrinsic limitations in reflecting the true histopathological status of the pulp. Further research is therefore needed to develop reproducible, evidence-based diagnostic and restorative protocols for VPT.

## 4 Conclusions

The maintenance of pulp vitality, absence of symptoms, and radiographic stability observed over 3 years suggest that biologically oriented VPT combined with contemporary adhesive restorative management may represent a feasible minimally invasive option in selected cases. Within the limitations of this three-case series, the hCSC Biodentine XP appeared clinically compatible with both immediate and staged restorative workflows. However, these findings should be interpreted as preliminary clinical evidence, and further studies are needed to confirm the long-term reproducibility and broader applicability of this approach.

## 5 Clinical relevance

The combination of Biodentine XP with universal adhesives and composite restorations may offer a practical bioactive–adhesive strategy for preserving pulp vitality while ensuring stable coronal sealing in both single- and two-step VPT protocols.

## Data Availability

The original contributions presented in the study are included in the article/Supplementary Material, further inquiries can be directed to the corresponding author.

## References

[1] Smith AJ Duncan HF Diogenes A Simon S Cooper PR. Exploiting the bioactive properties of the dentin-pulp Complex in regenerative endodontics. J Endod. (2016) 42:47–56. 10.1016/j.joen.2015.10.019 26699924

[2] Aguilar P Linsuwanont P. Vital pulp therapy in vital permanent teeth with cariously exposed pulp: a systematic review. J Endod. (2011) 37:581–7. 10.1016/j.joen.2010.12.004 21496652

[3] Duncan HF. Present status and future directions-vital pulp treatment and pulp preservation strategies. Int Endod J. (2022) 55(Suppl 3):497–511. 10.1111/iej.13688 35080024 PMC9306596

[4] Hirschberg CS Bogen G Galicia JC Lemon RR Peters OA Ruparel NB. AAE Position statement on vital pulp therapy. J Endod (2021) 47:1340–4. 10.1016/j.joen.2021.07.015 34352305

[5] Duncan HF Galler KM Tomson PL Simon S El-Karim I Kundzina R. European Society of Endodontology position statement: management of deep caries and the exposed pulp. Int Endod J. (2019) 52:923–34. 10.1111/iej.13080 30664240

[6] Komabayashi T Zhu Q Eberhart R Imai Y. Current status of direct pulp-capping materials for permanent teeth. Dent Mater J. (2016) 35:1–12. 10.4012/dmj.2015-013 26830819

[7] Ricucci D Loghin S Lin LM Spångberg LSW Tay FR. Is hard tissue formation in the dental pulp after the death of the primary odontoblasts a regenerative or a reparative process? J Dent. (2014) 42:1156–70. 10.1016/j.jdent.2014.06.012 25008021

[8] Ricucci D Siqueira JF Li Y Tay FR. Vital pulp therapy: histopathology and histobacteriology-based guidelines to treat teeth with deep caries and pulp exposure. J Dent. (2019) 86:41–52. 10.1016/j.jdent.2019.05.022 31121241

[9] Camilleri J Atmeh A Li X Meschi N. Present status and future directions: hydraulic materials for endodontic use. Int Endod J. (2022) 55(Suppl 3):710–77. 10.1111/iej.13709 35167119 PMC9314068

[10] Aminoshariae A Primus C Kulild JC. Tricalcium silicate cement sealers: do the potential benefits of bioactivity justify the drawbacks? J Am Dent Assoc. (2022) 153:750–60. 10.1016/j.adaj.2022.01.004 35260235

[11] Parirokh M Torabinejad M Dummer PMH. Mineral trioxide aggregate and other bioactive endodontic cements: an updated overview - part I: vital pulp therapy. Int Endod J. (2018) 51:177–205. 10.1111/iej.12841 28836288

[12] Duarte MAH Marciano MA Vivan RR Tanomaru Filho M Tanomaru JMG Camilleri J. Tricalcium silicate-based cements: properties and modifications. Braz Oral res. (2018) 32:e70. 10.1590/1807-3107bor-2018.vol32.0070 30365611

[13] About I. Biodentine: from biochemical and bioactive properties to clinical applications. G Ital Endod. (2016) 30:81–8. 10.1016/j.gien.2016.09.002

[14] Dawood AE Parashos P Wong RHK Reynolds EC Manton DJ. Calcium silicate-based cements: composition, properties, and clinical applications. J Investig Clin Dent. (2017) 8:e12195. 10.1111/jicd.12195 26434562

[15] Rajasekharan S Martens LC Cauwels RGEC Anthonappa RP. BiodentineTM material characteristics and clinical applications: a 3 year literature review and update. Eur Arch Paediatr Dent. (2018) 19:1–22. 10.1007/s40368-018-0328-x 29372451

[16] Celiksoz O Irmak O. Delayed vs. Immediate placement of restorative materials over Biodentine and RetroMTA: a micro-shear bond strength study. BMC Oral Health. (2024) 24:130. 10.1186/s12903-024-03917-3 38273289 PMC10811922

[17] Hardan L Mancino D Bourgi R Alvarado-Orozco A Rodríguez-Vilchis LE Flores-Ledesma A. Bond Strength of Adhesive Systems to Calcium Silicate-Based Materials: A Systematic Review and Meta-Analysis of In Vitro Studies. Gels. (2022) 8:311. 10.3390/gels8050311 35621609 PMC9141246

[18] Deepa VL Dhamaraju B Bollu IP Balaji TS. Shear bond strength evaluation of resin composite bonded to three different liners: theraCal LC, biodentine, and resin-modified glass ionomer cement using universal adhesive: an *in vitro* study. J Conserv Dent. (2016) 19:166–70. 10.4103/0972-0707.178696 27099425 PMC4815547

[19] Tosco V Monterubbianesi R Montagna P Orilisi G Vitiello F Song H. Vital pulp therapy: a three-year clinical case series on regenerative potential and adhesive restoration strategies. Regenesis Repair Rehabilitation. (2026) 2:99–111. 10.1016/j.rerere.2026.01.005

[20] Chiodera G Orsini G Tosco V Monterubbianesi R Manauta J Devoto W. Essential lines: a simplified filling and modeling technique for direct posterior composite restorations. Int J Esthet Dent. (2021) 16:168–84. 33969973

[21] Monterubbianesi R Tosco V Sabbatini S Orilisi G Conti C Özcan M. How can different polishing timing influence methacrylate and dimethacrylate bulk fill composites? Evaluation of chemical and physical properties. BioMed Res Int. (2020) 2020:e1965818. 10.1155/2020/1965818 PMC7199574 32382532

[22] Tosco V Monterubbianesi R Orilisi G Sabbatini S Conti C Özcan M. Comparison of two curing protocols during adhesive cementation: can the step luting technique supersede the traditional one? Odontology. (2020) 109:433–9. 10.1007/s10266-020-00558-0 33128650 PMC7954706

[23] Alsubait S Aljarbou F. Biodentine or mineral trioxide Aggregate as direct pulp capping material in mature permanent teeth with carious exposure? A systematic review and meta-analysis. Oper Dent. (2021) 46:631–40. 10.2341/20-277-LIT 35507905

[24] Moretto C Kopper PMP Münchow EA Scarparo RK. Association between patient age and vital pulp therapy outcomes: a systematic review and meta-analysis of prognostic studies. Int Endod J. (2025) 58:809–32. 10.1111/iej.14223 40133774

[25] Uesrichai N Nirunsittirat A Chuveera P Srisuwan T Sastraruji T Chompu-Inwai P. Partial pulpotomy with two bioactive cements in permanent teeth of 6- to 18-year-old patients with signs and symptoms indicative of irreversible pulpitis: a noninferiority randomized controlled trial. Int Endod J. (2019) 52:749–59. 10.1111/iej.13071 30638262

[26] Hashem DF Foxton R Manoharan A Watson TF Banerjee A. The physical characteristics of resin composite-calcium silicate interface as part of a layered/laminate adhesive restoration. Dent Mater. (2014) 30:343–9. 10.1016/j.dental.2013.12.010 24418628

[27] Abdullah HA Al-Ibraheemi ZA Hanoon ZA Haider J. Evaluation of shear bond strength of resin-based composites to Biodentine with three types of seventh-generation bonding agents: an *in vitro* study. Int J Dent. (2022) 2022:2830299. 10.1155/2022/2830299 35942229 PMC9356874

[28] Komora P Vámos O Gede N Hegyi P Kelemen K Galvács A. Comparison of bioactive material failure rates in vital pulp treatment of permanent matured teeth - a systematic review and network meta-analysis. Sci Rep. (2024) 14:18421. 10.1038/s41598-024-69367-7 39117767 PMC11310317

[29] Chen C-L Chi C-W Lee C-Y Tsai Y-L Kasimayan U K.P.O. M. Effects of surface treatments of bioactive tricalcium silicate-based restorative material on the bond strength to resin composite. Dent Mater. (2024) 40:102–10. 10.1016/j.dental.2023.10.027 37919112

[30] Raina A Sawhny A Paul S Nandamuri S. Comparative evaluation of the bond strength of self-adhering and bulk-fill flowable composites to MTA Plus, Dycal, Biodentine, and TheraCal: an *in vitro* study. Restor Dent Endod. (2020) 45:e10. 10.5395/rde.2020.45.e10 32110539 PMC7030959

[31] Gomez-Sosa JF Granone-Ricella M Rosciano-Alvarez M Barrios-Rodriguez VD Goncalves-Pereira J Caviedes-Bucheli J. Determining factors in the success of direct pulp capping: a systematic review. J Contemp Dent Pract. (2024) 25:392–401. 10.5005/jp-journals-10024-3673 38956856

[32] Hernández-Cabanillas JC Hardan L Cuevas-Suárez CE Olivares-Acosta I Dang AT Tosco V. Comparative evaluation of the physicochemical and biological properties of calcium silicate-based pulp capping materials. Front Dent Med. (2025) 6:1737941. 10.3389/fdmed.2025.1737941 41458174 PMC12741156

