# Supplementary material for: Vital pulp therapy using Biodentine XP and adhesive restorations: a three-year case series
Source: Front Dent Med. 2026 Jul 29;7:1837786. doi: 10.3389/fdmed.2026.1837786 (PMC13461418; doi:10.3389/fdmed.2026.1837786)
Supplement: Supplementary file 1 [file Table1.docx]

**CARE Checklist**

*Based on the revised manuscript: “Vital Pulp Therapy Using Biodentine XP and Adhesive Restorations: A Three-Year Case Series”*

Page references correspond to the revised manuscript pagination.

| **Topic** | **Item** | **Checklist item description** | **Reported on page** |
| --- | --- | --- | --- |
| Title | 1 | The diagnosis or intervention of primary focus followed by the words “case report”. | 1 |
| Key Words | 2 | 2 to 5 key words that identify diagnoses or interventions in this case report, including “case report”. | 1 |
| Abstract | 3a | Introduction: What is unique about this case and what does it add to the scientific literature? | 1 |
| Abstract | 3b | Main symptoms and/or important clinical findings. | 1 |
| Abstract | 3c | The main diagnoses, therapeutic interventions, and outcomes. | 1–2 |
| Abstract | 3d | Conclusion—What is the main “take-away” lesson(s) from this case? | 2 |
| Introduction | 4 | One or two paragraphs summarizing why this case is unique (may include references). | 2 |
| Patient Information | 5a | De-identified patient specific information. | 3–10 |
| Patient Information | 5b | Primary concerns and symptoms of the patient. | 3–10 |
| Patient Information | 5c | Medical, family, and psycho-social history including relevant genetic information. | 3 (medical history); family/psychosocial/genetic: N/A |
| Patient Information | 5d | Relevant past interventions with outcomes. | 8–10 |
| Clinical Findings | 6 | Describe significant physical examination (PE) and important clinical findings. | 3–10 |
| Timeline | 7 | Historical and current information from this episode of care organized as a timeline. | 3–10; Table 1 |
| Diagnostic Assessment | 8a | Diagnostic testing (such as PE, laboratory testing, imaging, surveys). | 3–10 |
| Diagnostic Assessment | 8b | Diagnostic challenges (such as access to testing, financial, or cultural). | N/A |
| Diagnostic Assessment | 8c | Diagnosis (including other diagnoses considered). | 3–10 |
| Diagnostic Assessment | 8d | Prognosis (such as staging in oncology) where applicable. | N/A |
| Therapeutic Intervention | 9a | Types of therapeutic intervention (such as pharmacologic, surgical, preventive, self-care). | 4–10 |
| Therapeutic Intervention | 9b | Administration of therapeutic intervention (such as dosage, strength, duration). | 4–10 |
| Therapeutic Intervention | 9c | Changes in therapeutic intervention (with rationale). | 6, 8–10 |
| Follow-up and Outcomes | 10a | Clinician and patient-assessed outcomes (if available). | 3–10, 12 |
| Follow-up and Outcomes | 10b | Important follow-up diagnostic and other test results. | 4–10, 12 |
| Follow-up and Outcomes | 10c | Intervention adherence and tolerability (How was this assessed?). | N/A |
| Follow-up and Outcomes | 10d | Adverse and unanticipated events. | N/A |
| Discussion | 11a | A scientific discussion of the strengths AND limitations associated with this case report. | 10–12 |
| Discussion | 11b | Discussion of the relevant medical literature with references. | 10–12 |
| Discussion | 11c | The scientific rationale for any conclusions (including assessment of possible causes). | 10–12 |
| Discussion | 11d | The primary “take-away” lessons of this case report (without references) in a one paragraph conclusion. | 12 |
| Patient Perspective | 12 | The patient should share their perspective in one to two paragraphs on the treatment(s) they received. | N/A |
| Informed Consent | 13 | Did the patient give informed consent? Please provide if requested. | Yes; page 3 |

Note: Items not applicable to the present case series were marked as N/A.
