# Supplementary material for: Vital pulp therapy using Biodentine XP and adhesive restorations: a three-year case series
Source: Front Dent Med. 2026 Jul 29;7:1837786. doi: 10.3389/fdmed.2026.1837786 (PMC13461418; doi:10.3389/fdmed.2026.1837786)
Supplement: Supplementary file 2 [file Table2.docx]

Supplementary Table S2. Modified USPHS-based criteria used for restoration survival assessment.

| **Domain** | **Definition** | **Score A** | **Score B** | **Score C** |
| --- | --- | --- | --- | --- |
| Marginal adaptation | Continuity and clinical adaptation of restoration margins | Clinically acceptable margin integrity | Clinically unacceptable but not requiring replacement | Clinically unacceptable and requiring replacement |
| Marginal discoloration | Presence of discoloration along restoration margins | Absent or clinically acceptable | Present but not requiring replacement | Present and requiring replacement |
| Secondary caries | Presence of recurrent caries adjacent to the restoration | Absent | Suspected/limited, not requiring replacement | Present and requiring replacement |
| Retention/fracture events | Loss of restoration or structural fracture affecting restoration integrity | Absent | Minor defect not requiring replacement | Failure requiring replacement |
