# Supplementary material for: Vital pulp therapy using Biodentine XP and adhesive restorations: a three-year case series
Source: Front Dent Med. 2026 Jul 29;7:1837786. doi: 10.3389/fdmed.2026.1837786 (PMC13461418; doi:10.3389/fdmed.2026.1837786)
Supplement: Supplementary file 3 [file Table3.docx]

Supplementary Table S3. Restoration survival outcomes of the treated teeth at 3-year follow-up.

| **Case** | **Tooth** | **Restorative approach** | **Marginal adaptation** | **Marginal discoloration** | **Secondary caries** | **Retention/fracture events** | **Overall restorative status** |
| --- | --- | --- | --- | --- | --- | --- | --- |
| 1 | Mandibular second premolar | Direct composite restoration over Biodentine XP | A | A | A | A | Clinically acceptable |
| 2 | Mandibular first molar | Immediate direct composite restoration over Biodentine XP | A | A | A | A | Clinically acceptable |
| 3a | Mandibular second molar | Indirect overlay over retained Biodentine XP base | A | A | A | A | Clinically acceptable |
| 3b | Mandibular third molar | Direct composite restoration over retained Biodentine XP base | A | A | A | A | Clinically acceptable |

*A = clinically acceptable; B = clinically unacceptable but not requiring replacement; C = clinically unacceptable and requiring replacement.*
